# Supplementary material for: Chemical Constituents from Fraxinus hupehensis and Their Antifungal and Herbicidal Activities
Source: Biomolecules. 2020 Jan 2;10(1):74. doi: 10.3390/biom10010074 (PMC7022268; doi:10.3390/biom10010074)

Supplementary material

Chemical constituents from *Fraxinus hupehensis* and their antifungal and herbicidal activities

Chi-Na Zhao,[a,b †] Zong-Li Yao,[a,b †] Dan Yang,[a, b] Jian Ke,[a,b] Qing-Lai Wu[*a,b], Jun-Kai Li [*a,b], Xu-Dong Zhou[*c]

[a] School of Agriculture, Yangtze University, Jingzhou, 434020, *P. R.* China.

[b] Institute of Pesticides, Yangtze University, Jingzhou, *P. R.* China.

[c] TCM and Ethnomedicine Innovation & Development Laboratory, School of Pharmacy, Hunan University of Chinese Medicine, Changsha, Hunan 410208, *P. R.* China.

[†] Chi-Na Zhao and Zong-Li Yao contributed equally to this study.

Corresponding author email:
wql106@163.com (Qing-Lai Wu);
junkaili@sina.com (Jun-Kai Li);
xudongzhou999@163.com (Xu-Dong Zhou).

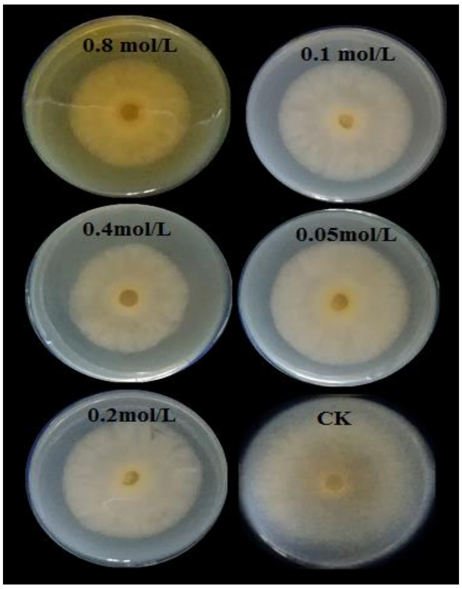

Figure S1. Antifungal activity of **2** against *Bipolaris maydis* evaluated by the mycelium growth rate method

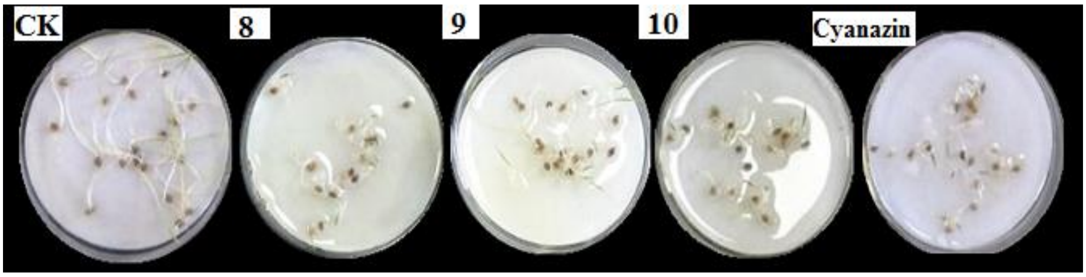

Figure S2. Comparison of the effects of **8–10** and Cyanazine against *Echinochloa crus-galli* growth(CK reprents *Echinochloa crus-galli* grown on regular medium.)

Spectra of compounds

Spectra of compound 1

^{1}H NMR spectrum

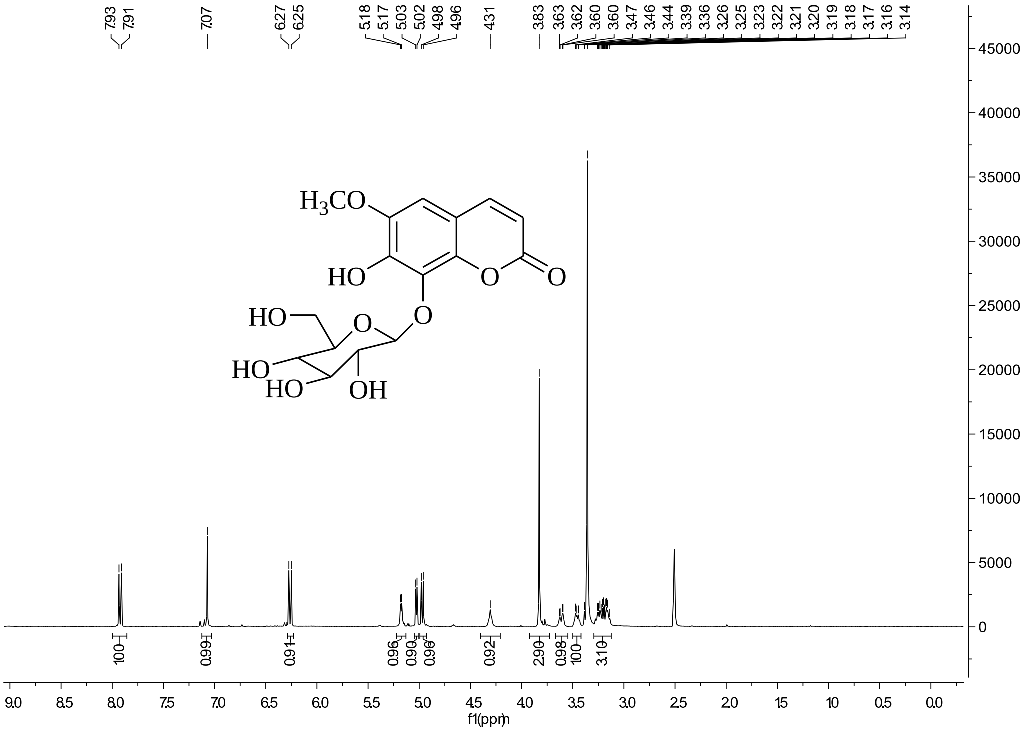

Spectra of compound 1

^{13}C NMR spectrum

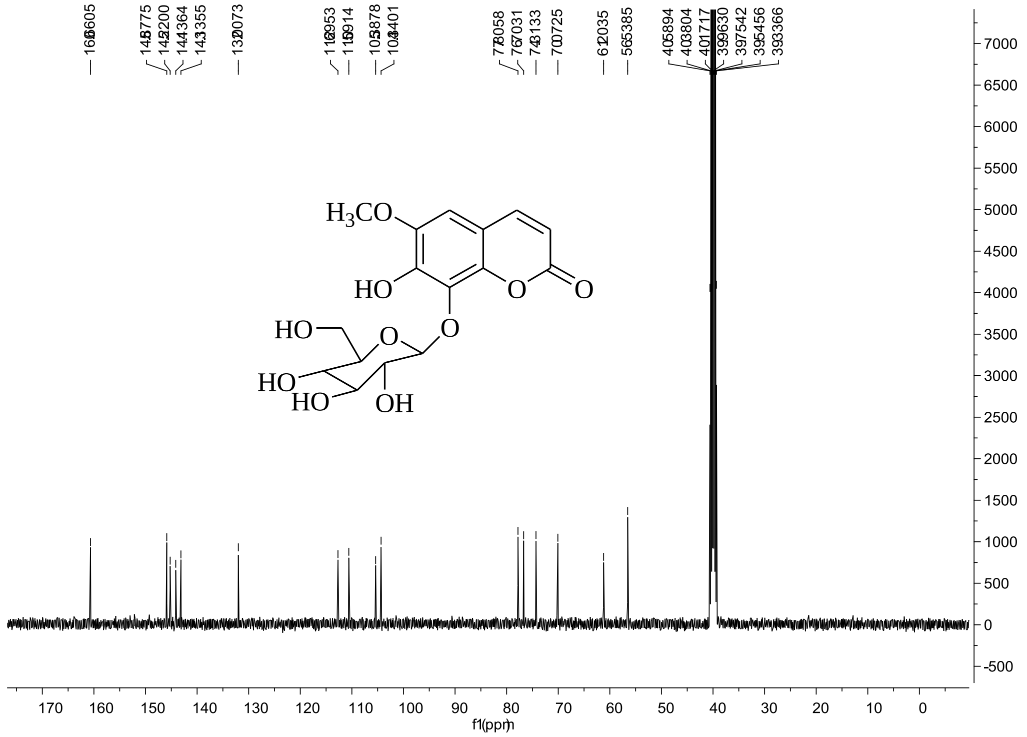

Spectra of compound 1

HRMS spectrum

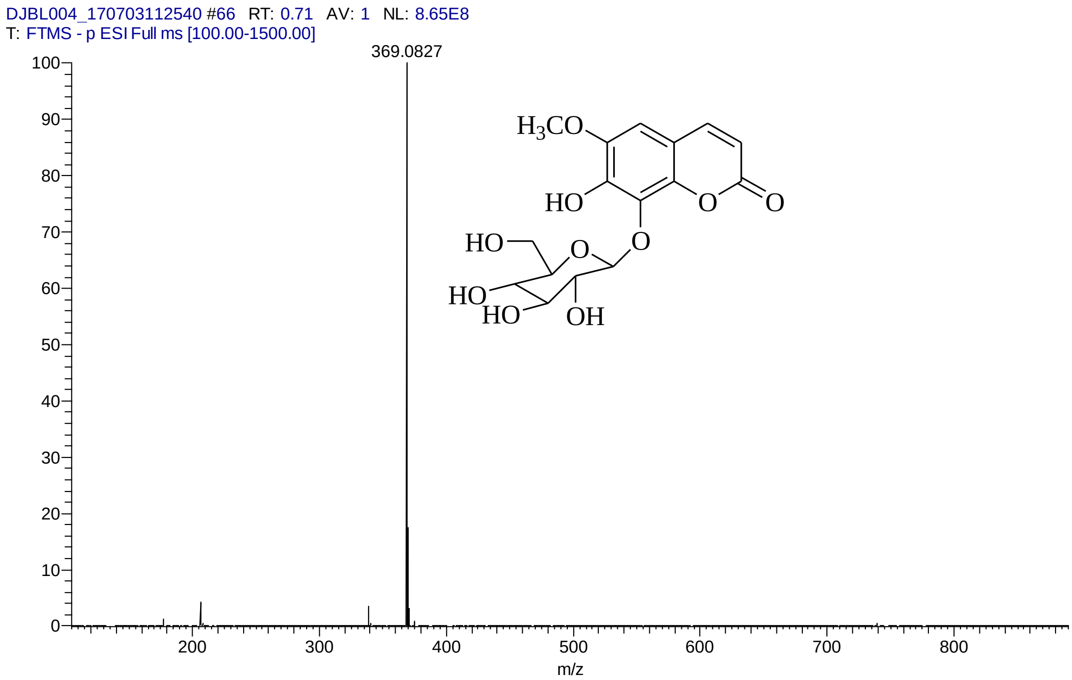

Spectra of compound 2

¹H NMR spectrum

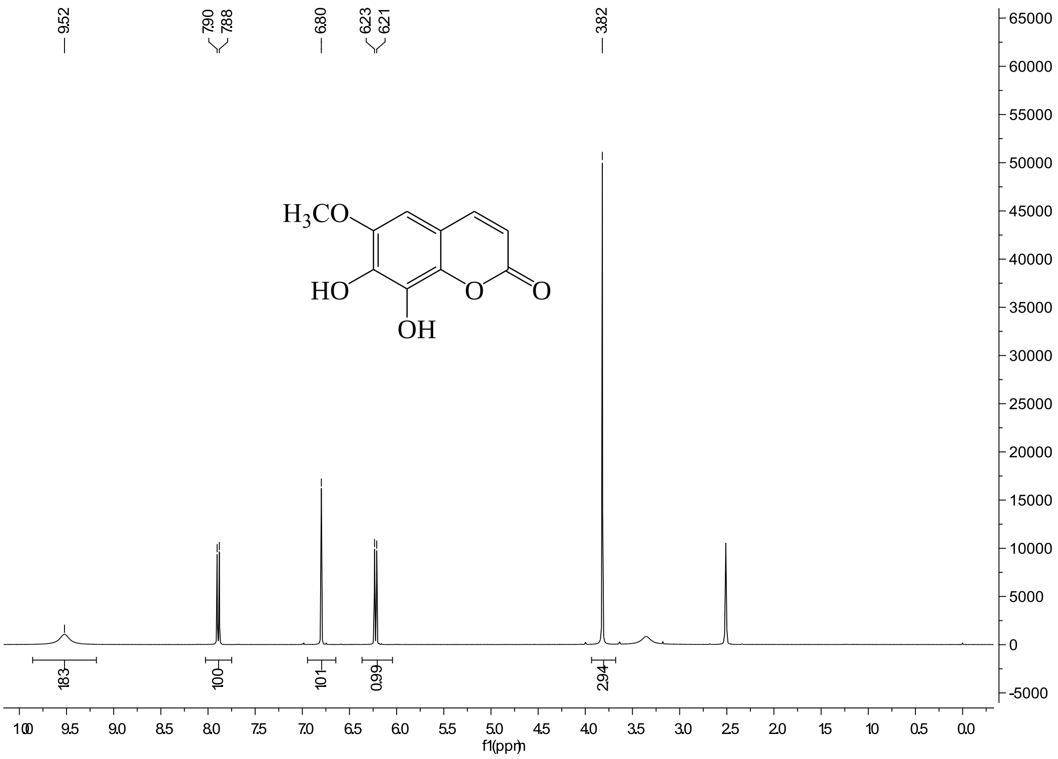

Spectra of compound 2

^{13}C NMR spectrum

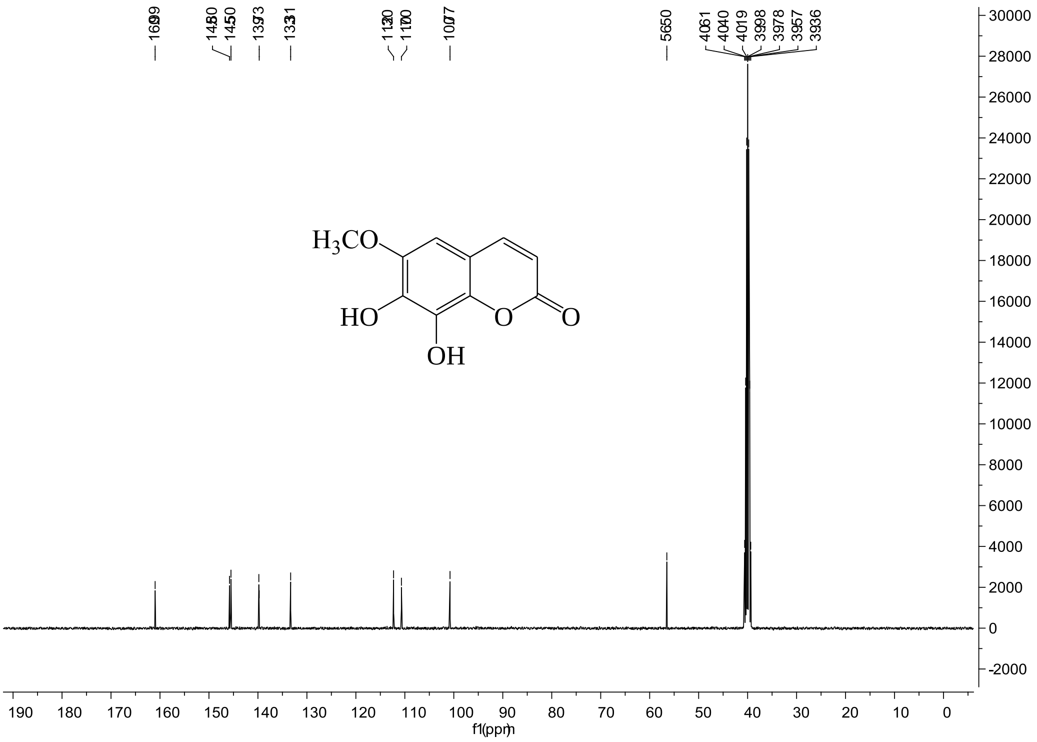

Spectra of compound 2

HRMS spectrum

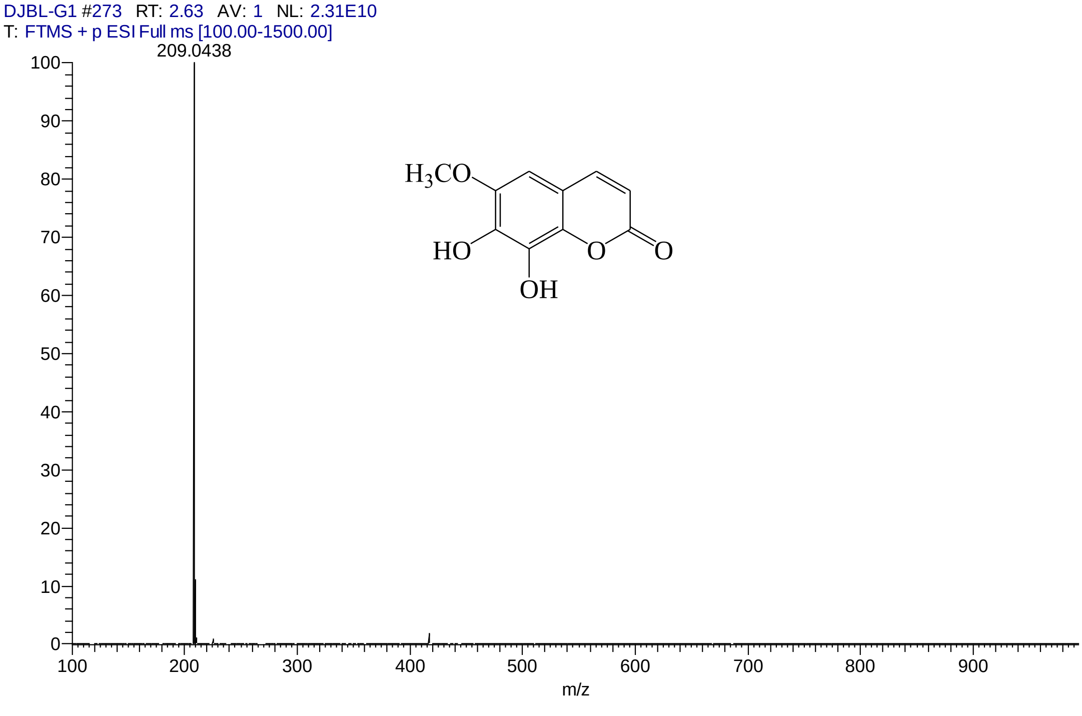

Spectra of compound 3

^{1}H NMR spectrum

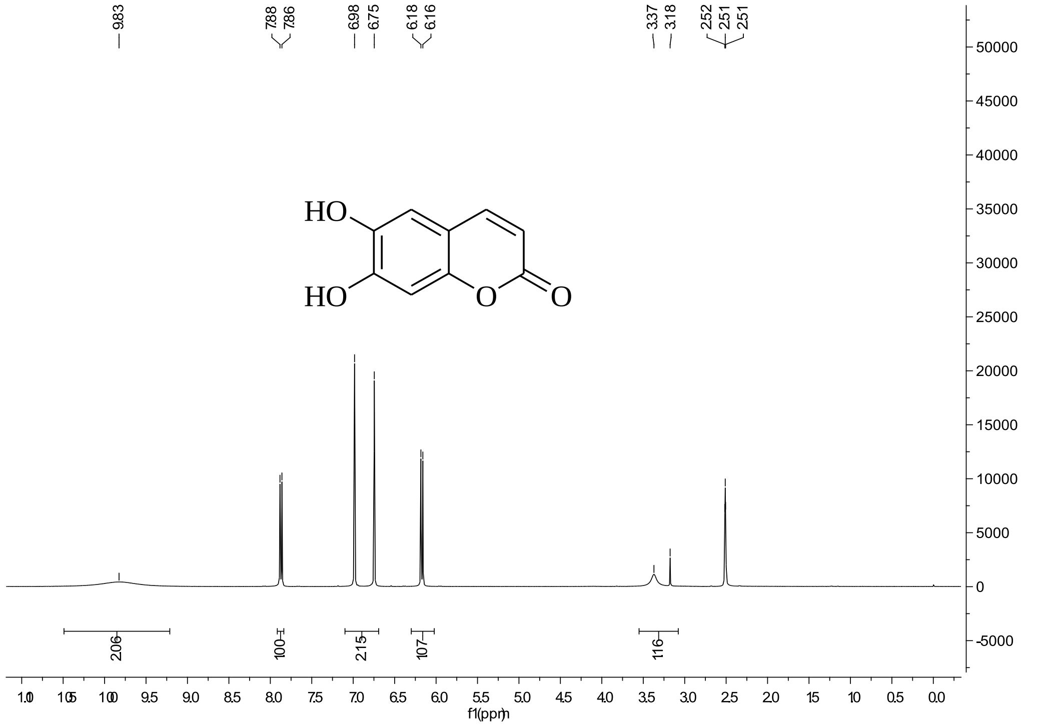

Spectra of compound 3

^{13}C NMR spectrum

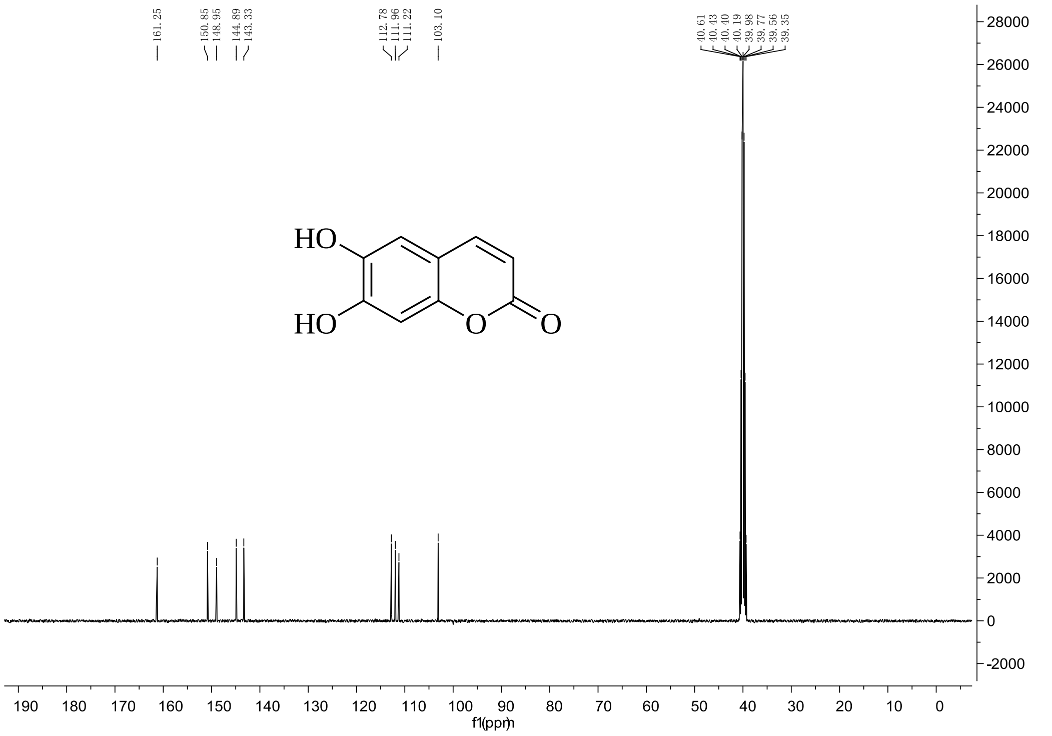

Spectra of compound 3

HRMS spectrum

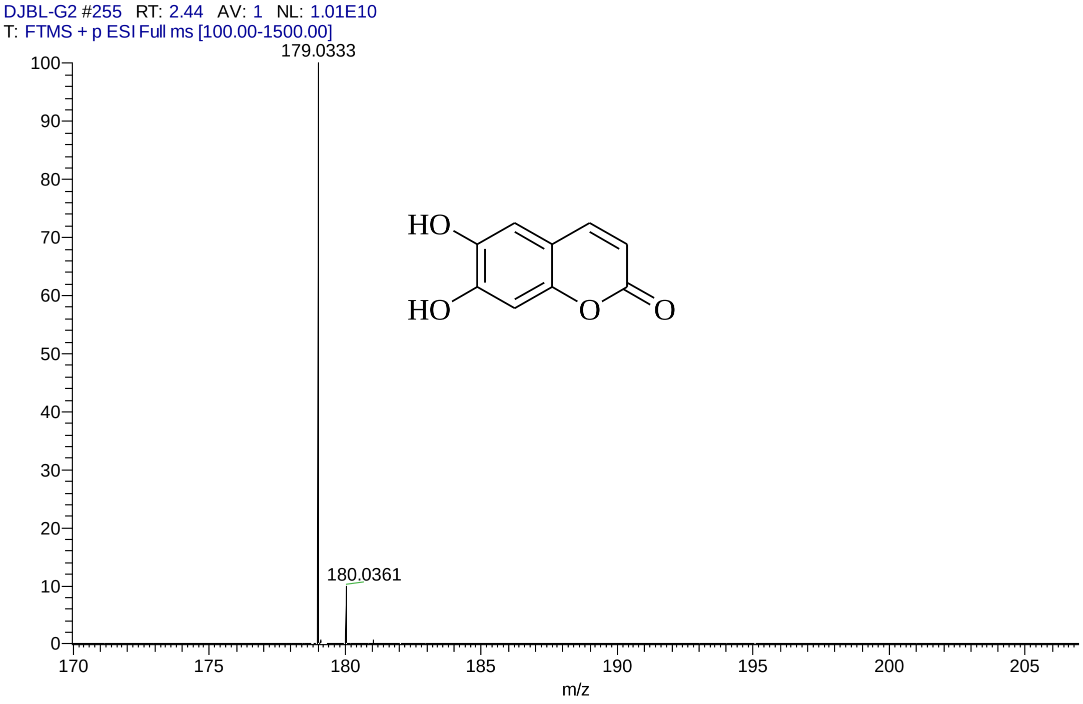

Spectra of compound 4

^{1}H NMR spectrum

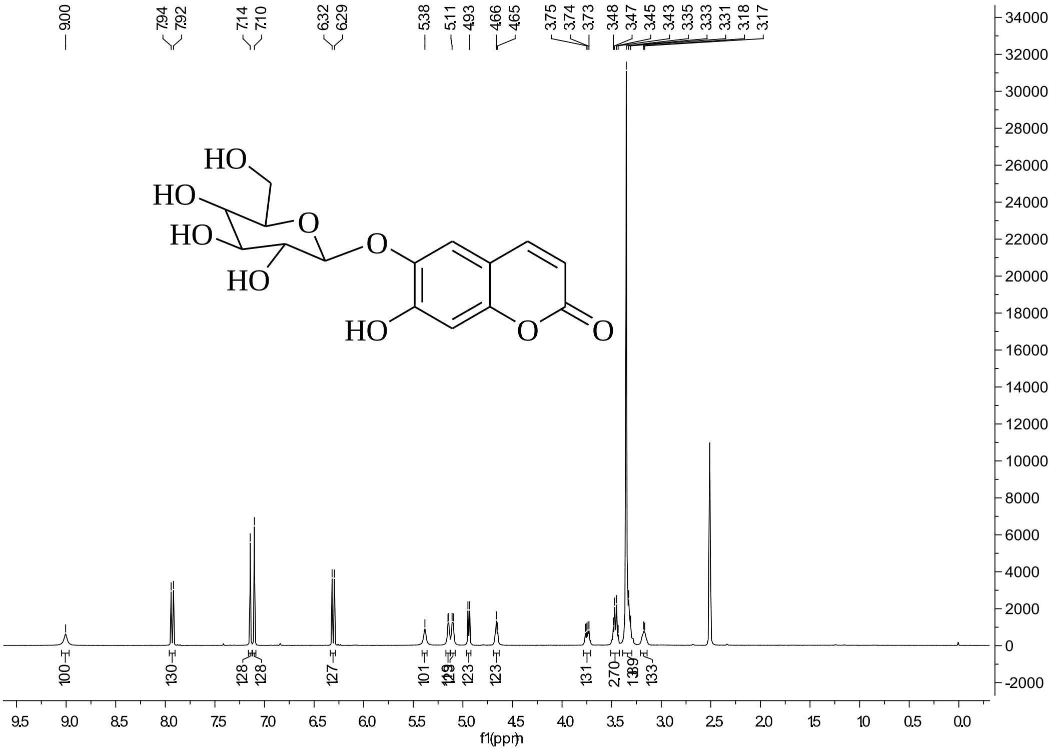

Spectra of compound 4

^{13}C NMR spectrum

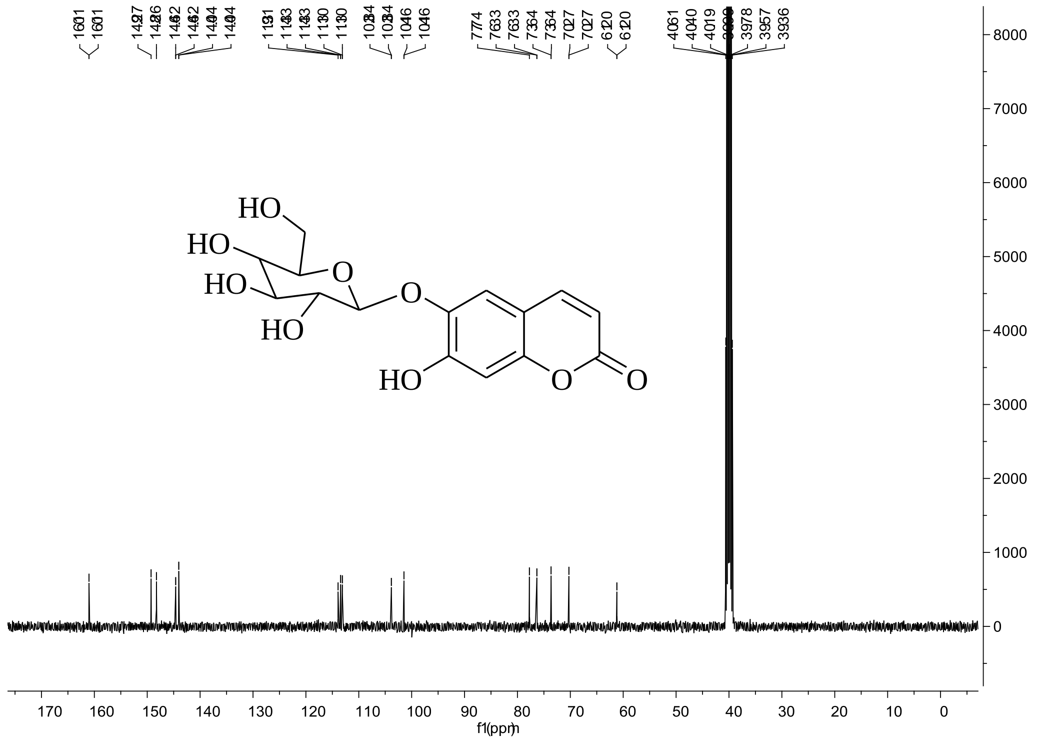

Spectra of compound 4

HRMS spectrum

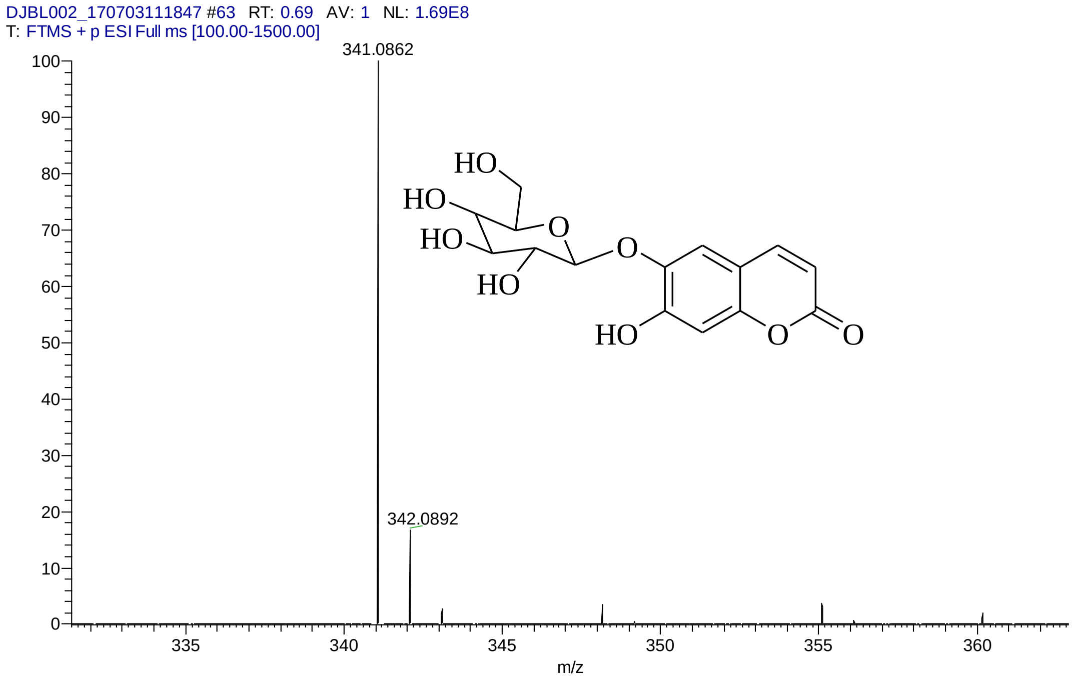

Spectra of compound 5

^{1}H NMR spectrum

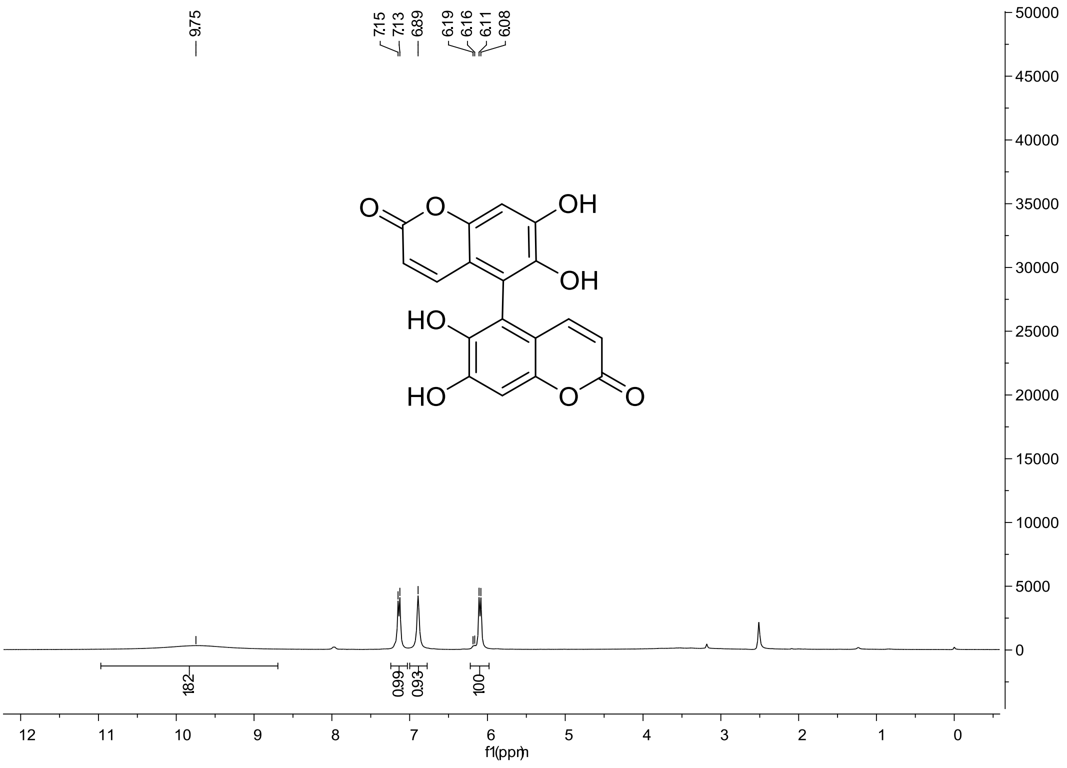

Spectra of compound 5

^{13}C NMR spectrum

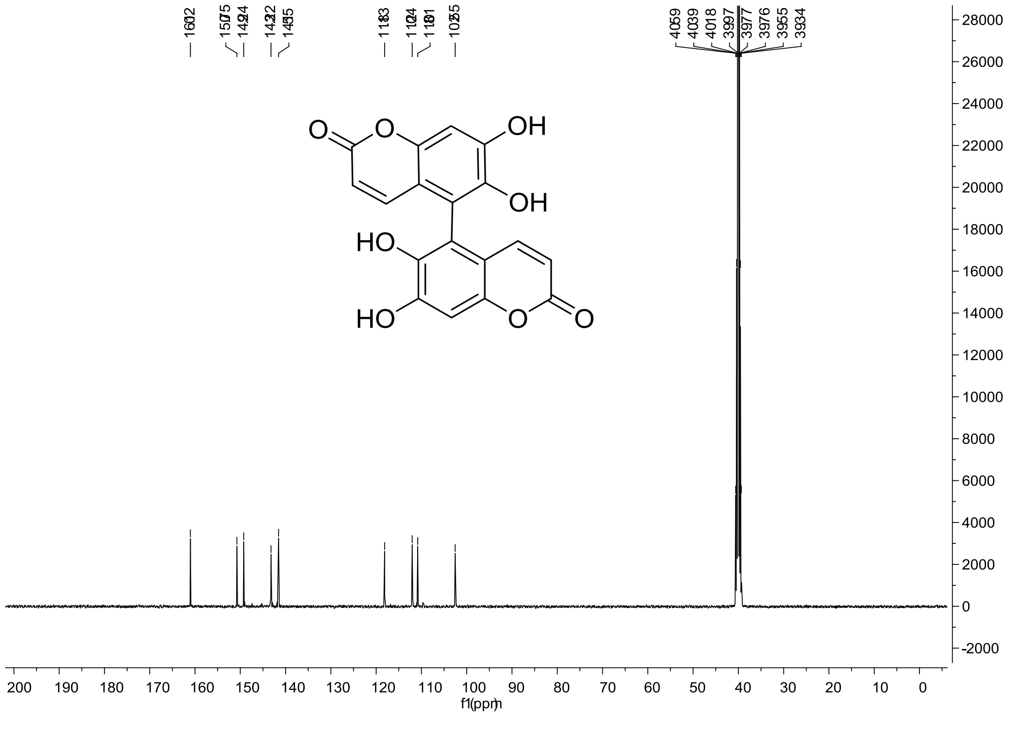

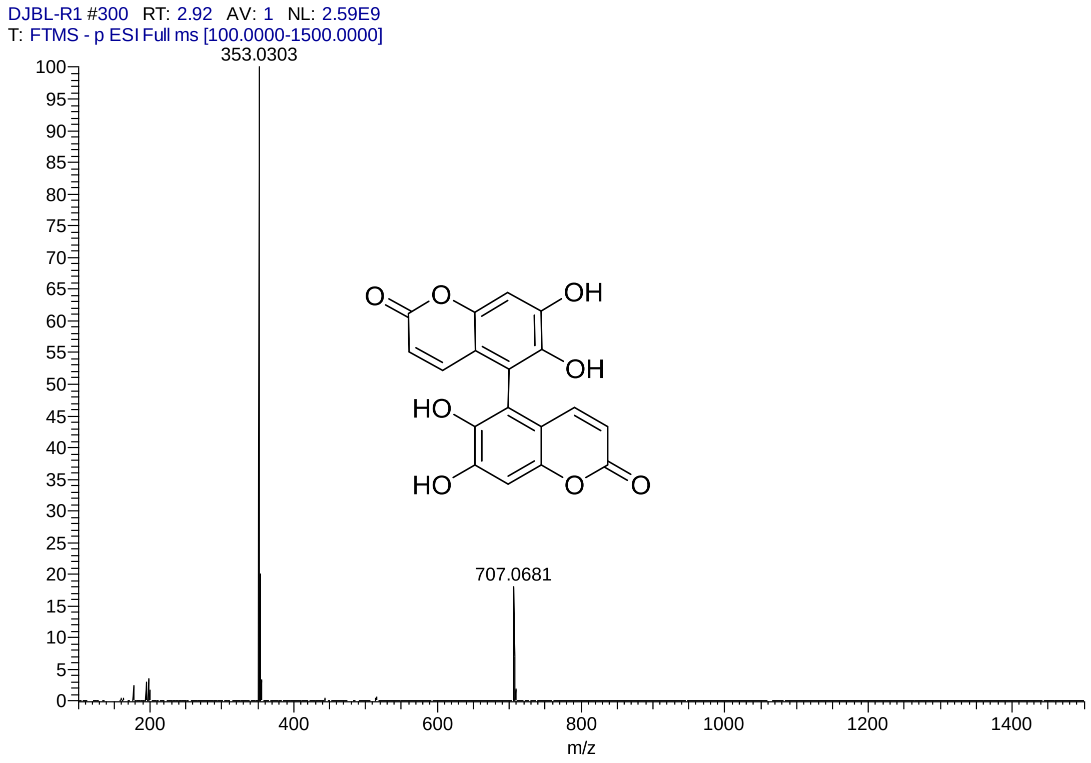

Spectra of compound 6

^{1}H NMR spectrum

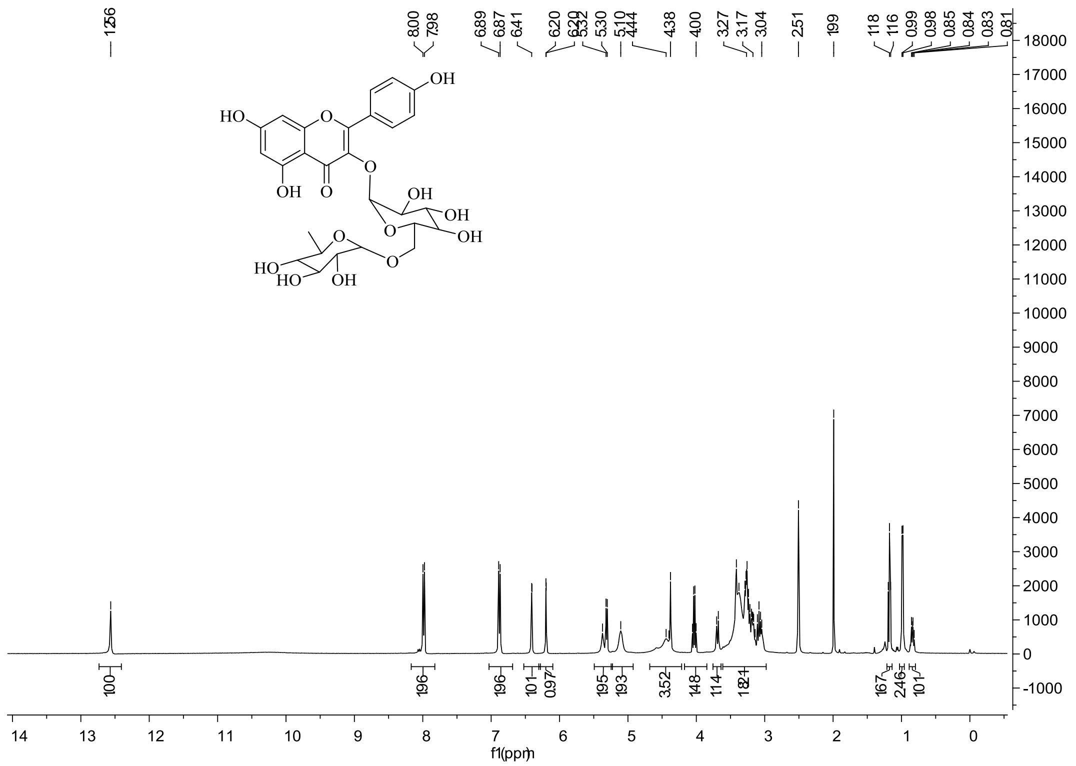

Spectra of compound 6

^{13}C NMR spectrum

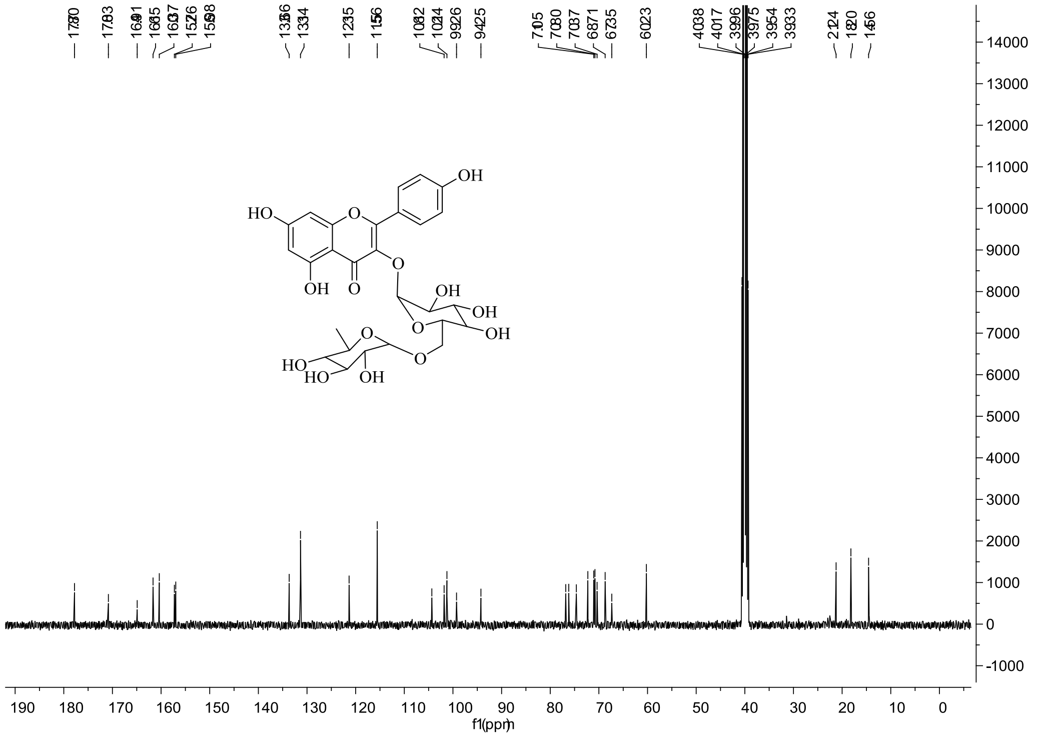

Spectra of compound 6

HRMS spectrum

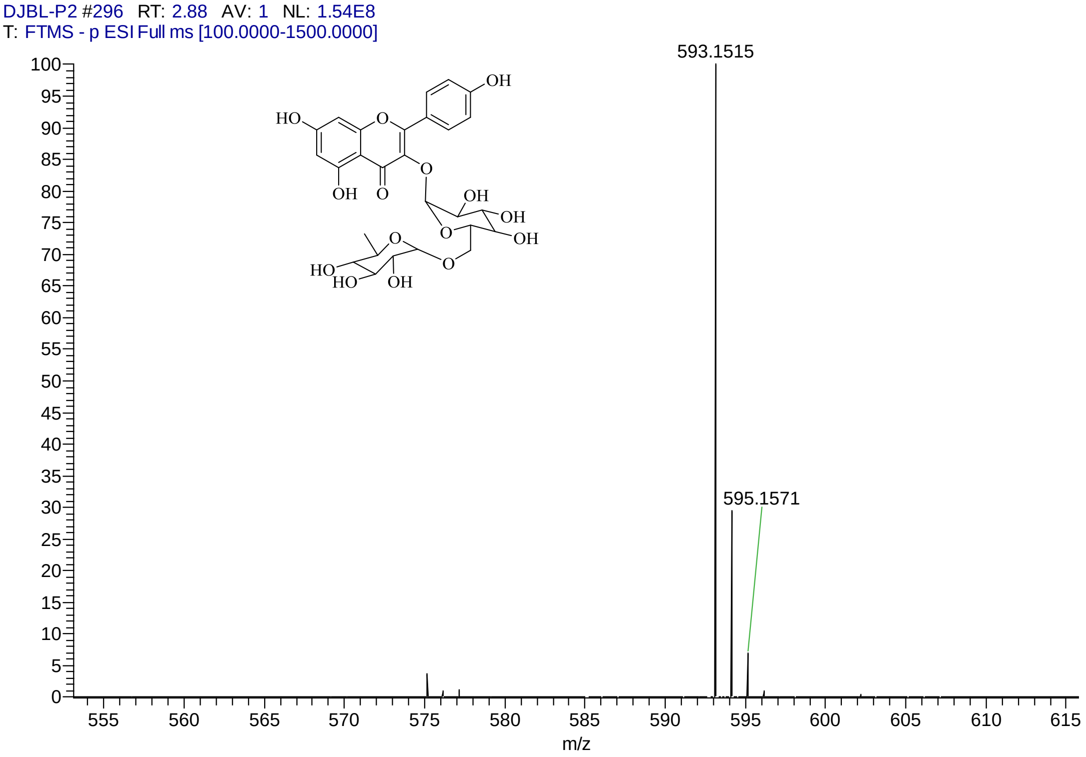

Spectra of compound 7

^{1}H NMR spectrum

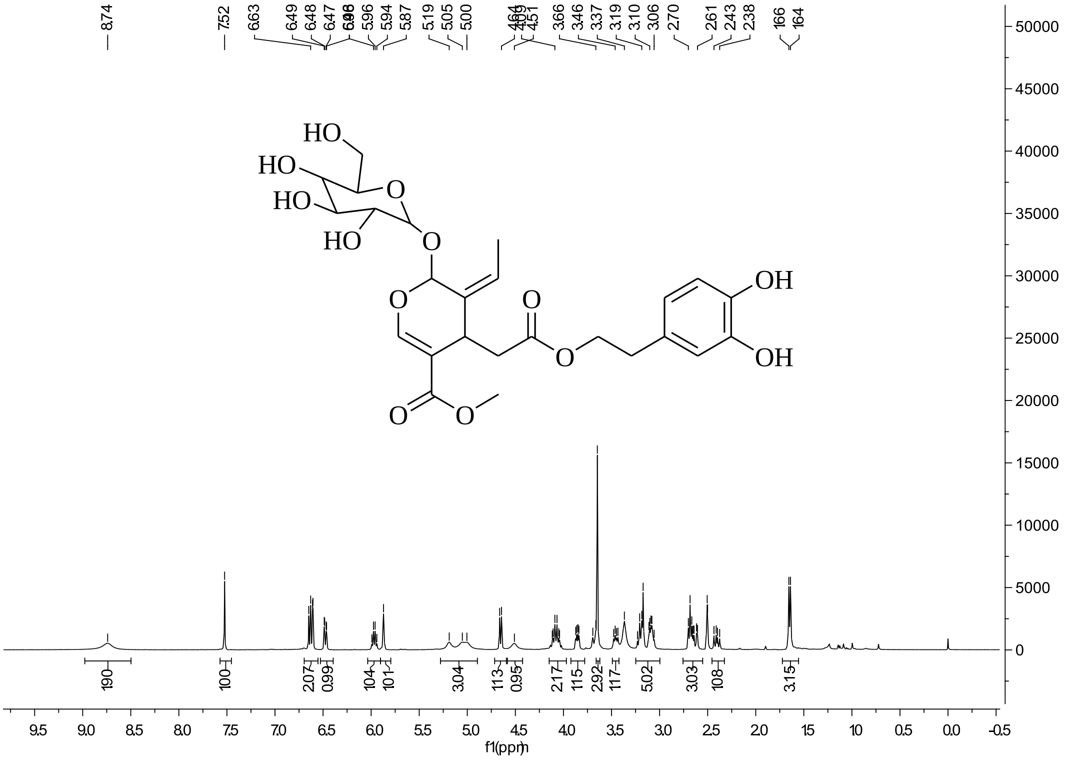

Spectra of compound 7

^{13}C NMR spectrum

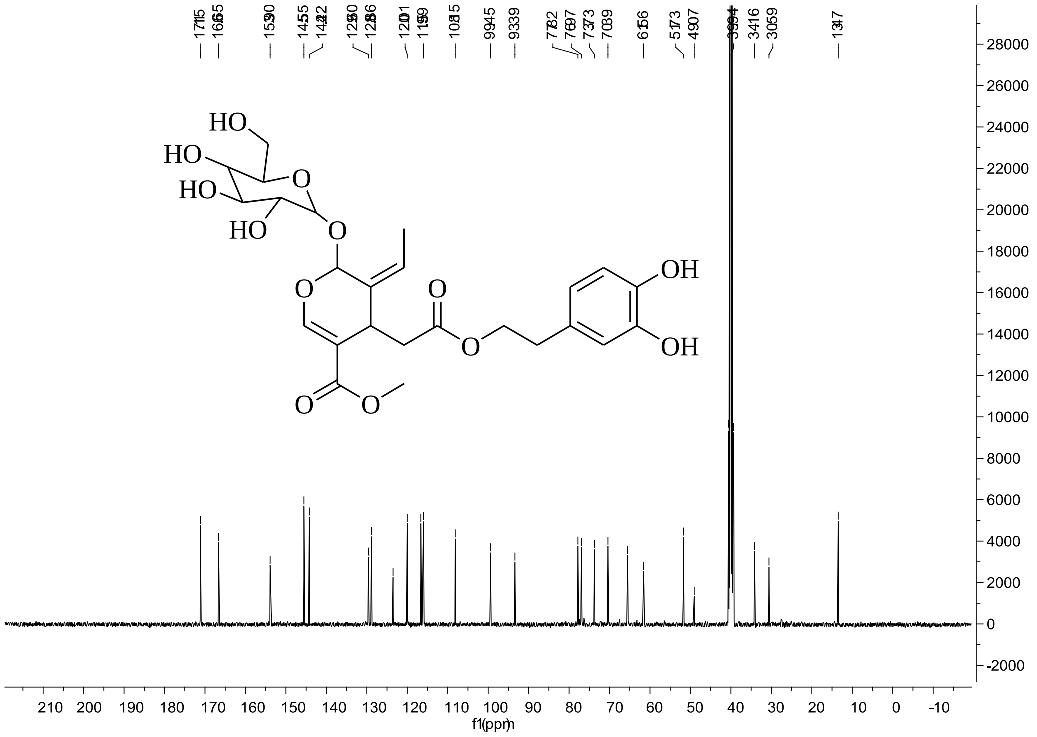

Spectra of compound 7

HRMS spectrum

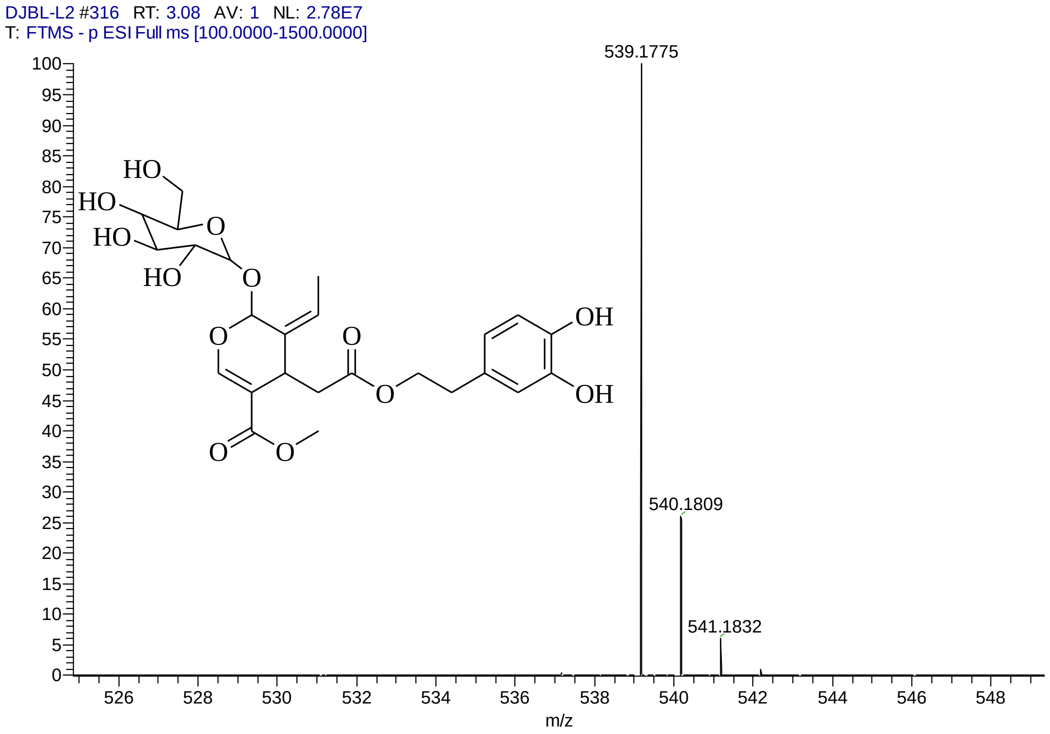

Spectra of compound 8

^{1}H NMR spectrum

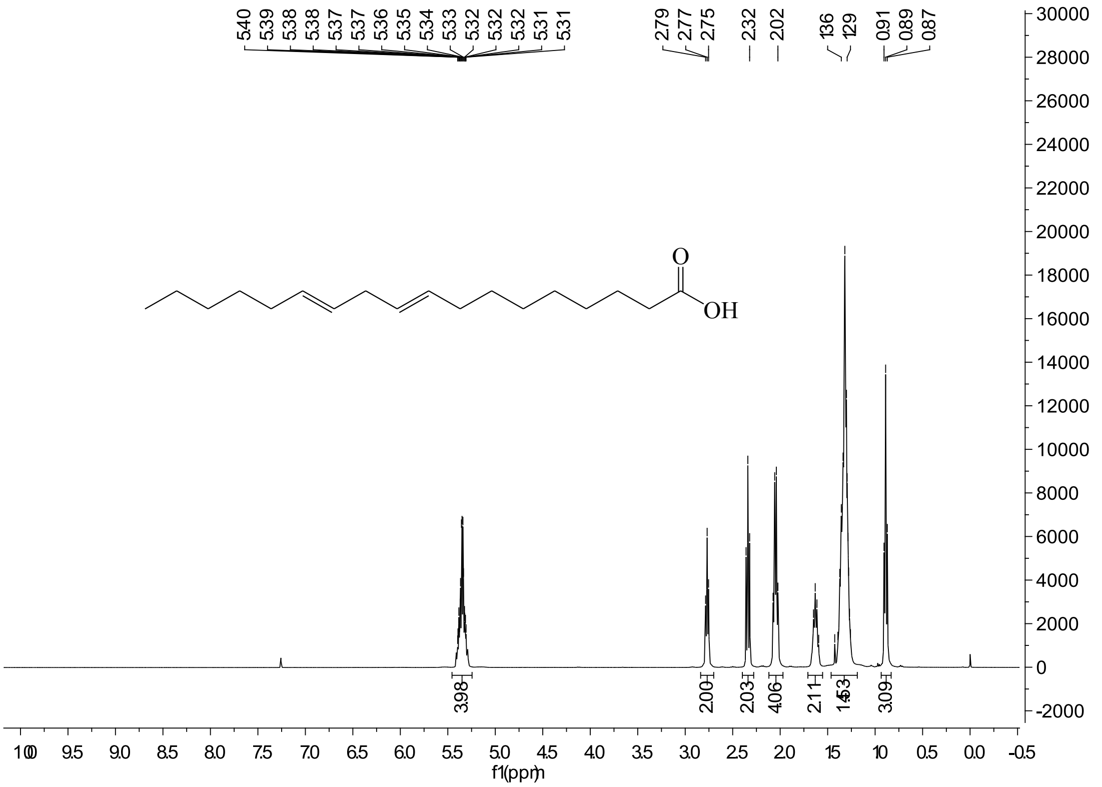

Spectra of compound 8

^{13}C NMR spectrum

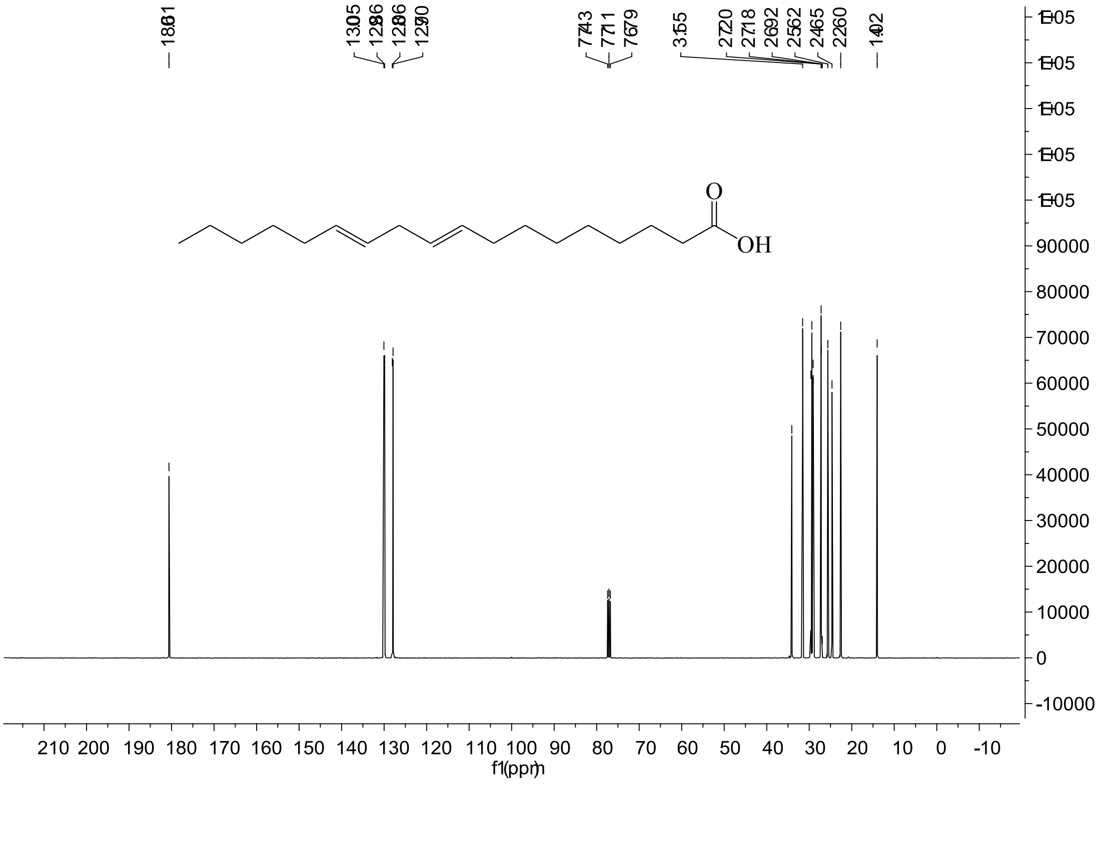

Spectra of compound 8

HRMS spectrum

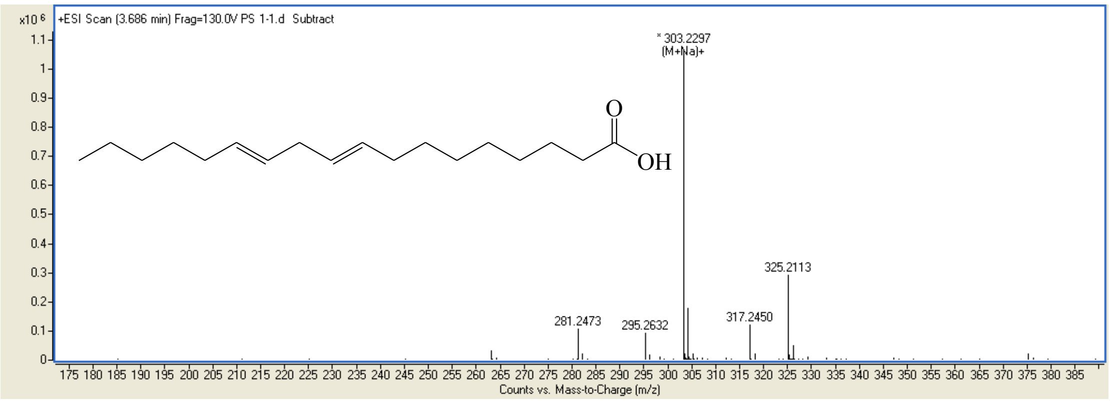

Spectra of compound 9

^{1}H NMR spectrum

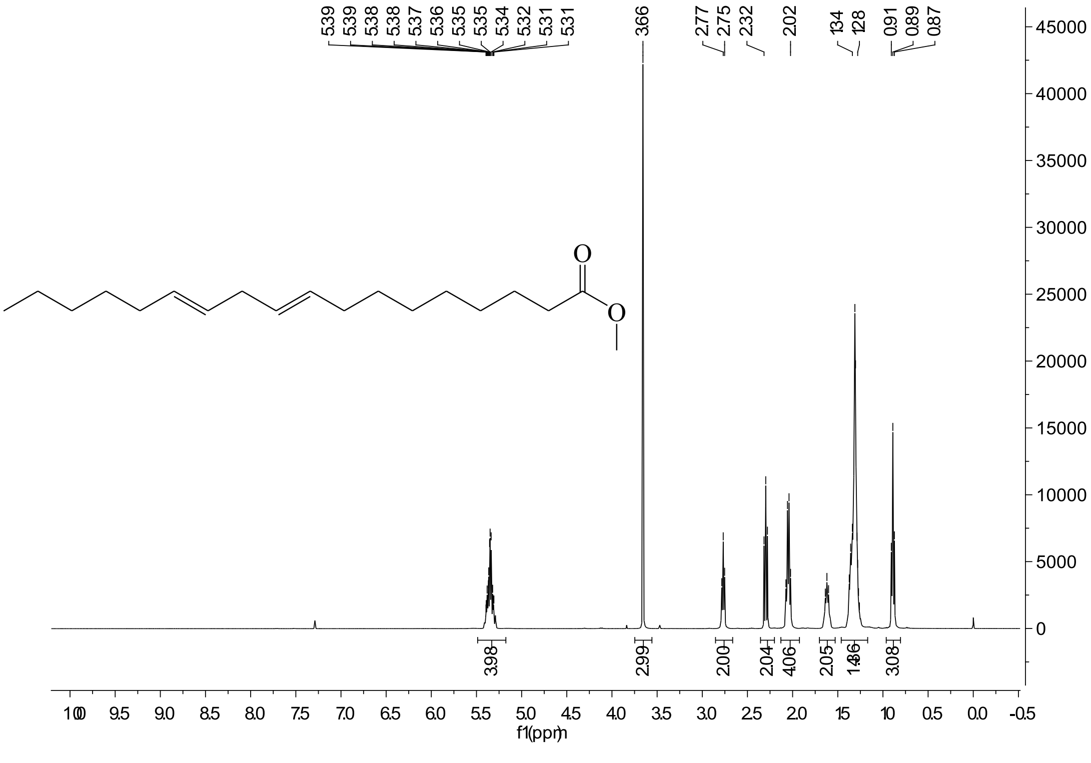

Spectra of compound 9

^{13}C NMR spectrum

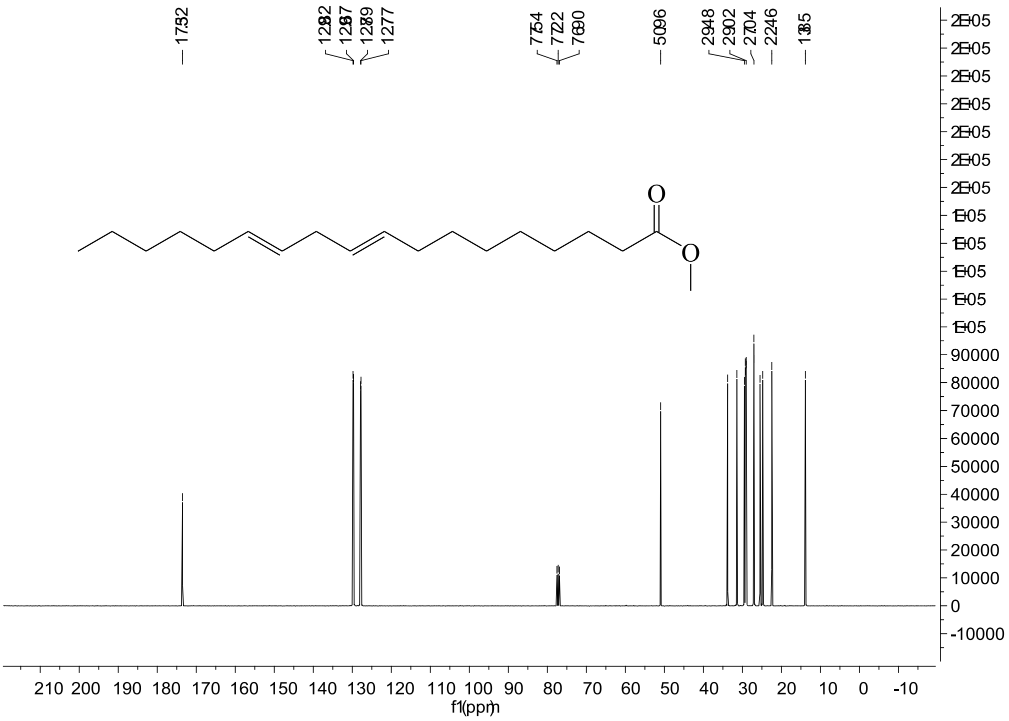

Spectra of compound 9

HRMS spectrum

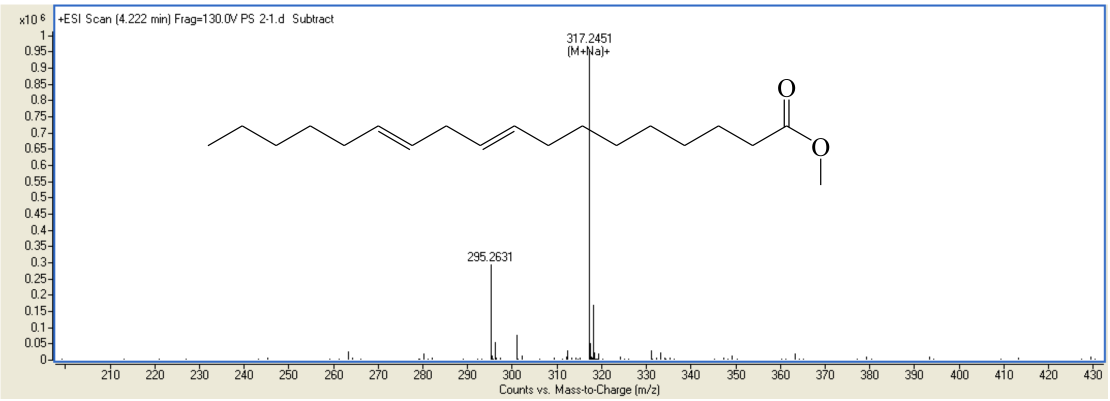

Spectra of compound 10

^{1}H NMR spectrum

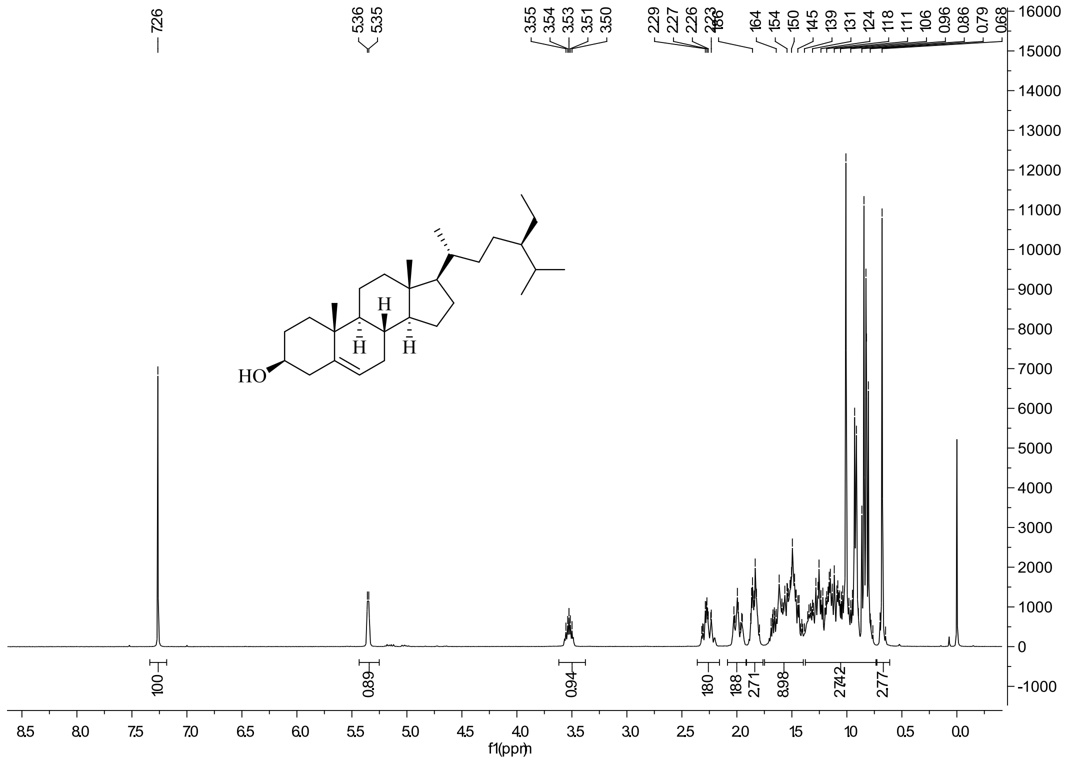

Spectra of compound 10

^{13}C NMR spectrum

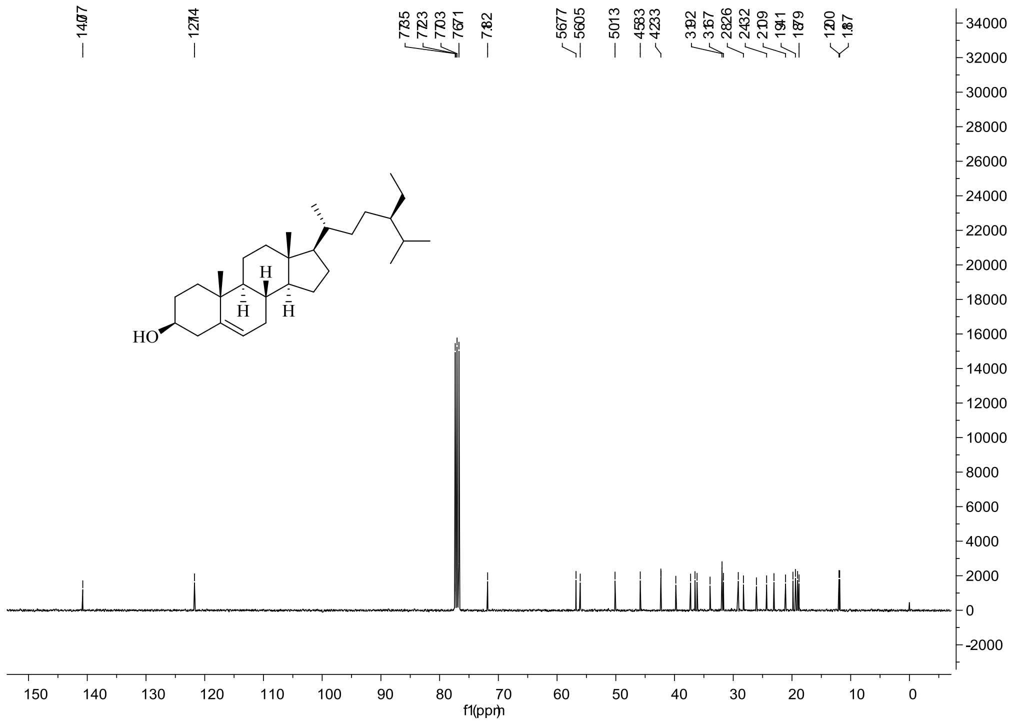

Spectra of compound 10

HRMS spectrum

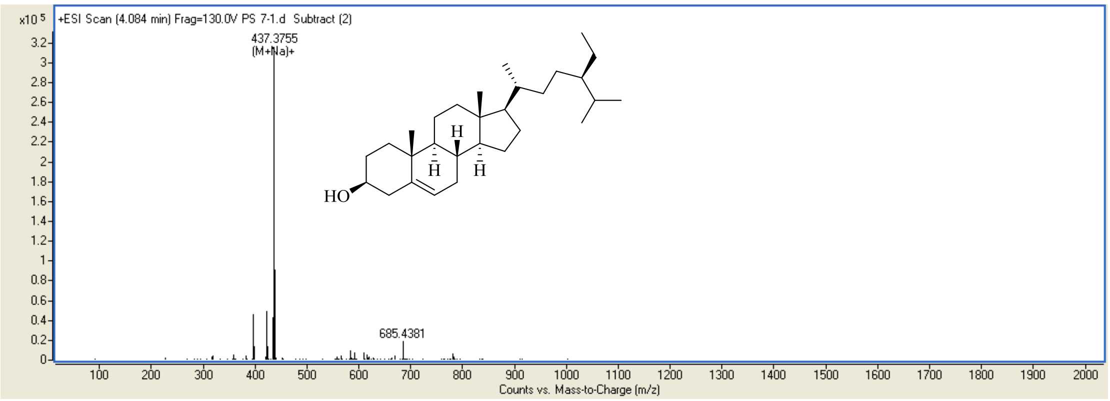

Supplement: Supplementary file 1 [file biomolecules-10-00074-s001.pdf]
